# Development and validation of a predictive model for pathological upgrading in colorectal polyps based on endoscopic forceps biopsy

**DOI:** 10.3389/fmed.2026.1748424

**Published:** 2026-02-13

**Authors:** Ziyao Cheng, Chang Zhang, Feng Yu

**Affiliations:** 1 Department of Oncology, The First Affiliated Hospital of Nanchang University, Nanchang, Jiangxi, China; 2 Department of Gastroenterology, The Fourth Affiliated Hospital of Anhui Medical University, Hefei, China

**Keywords:** colorectal polyps, nomogram, pathological upgrade, predictive model, risk factor

## Abstract

**Objectives:**

To develop and validate a model for predicting the risk of pathological upgrading in patients with colorectal polyps.

**Methods:**

This prospective study enrolled 616 patients who were diagnosed with colorectal polyps by endoscopic forceps biopsy at the Fourth Affiliated Hospital of Anhui Medical University from August 2022 to October 2025. After exclusion, 593 patients were included in the final analysis. They were randomly divided into a training cohort (*n* = 415) and a testing cohort (*n* = 178) at a ratio of 7:3. In the training cohort, least absolute shrinkage and selection operator (LASSO) regression was used to select possible predictive factors. Multivariable logistic regression was then applied to identify independent risk factors. A nomogram was developed to show the prediction model in a visual way. The performance of the model was assessed using the receiver operating characteristic (ROC) curve, calibration plot, Hosmer–Lemeshow goodness-of-fit test, and decision curve analysis (DCA). SHapley Additive Explanations (SHAP) were also used to help explain the model results.

**Results:**

The polyp was located in the rectum, with an MTD ≥ 30 mm. The polyp had a villous structure. Erosion of the polyp and redness of the polyp surface were identified as significant predictors of pathological escalation in patients with colorectal polyps. A nomogram developed based on these predictors showed excellent predictive performance. The area under the ROC curve (AUC) for the training set and the test set is 0.890 and 0.922, respectively. The calibration curve and the Hosmer-Lemeshow test show a high degree of consistency between the predicted and observed results, and DCA confirms that the model has superior clinical practicality.

**Conclusion:**

This study developed and validated a risk prediction model for pathological upgrade of colorectal polyps based on five endoscopic factors, including rectal location, maximum tumor diameter (MTD) ≥ 30 mm, villous structure, erosion, and a red surface color. The model serves as a practical clinical tool that allows endoscopists to assess patient risk with high accuracy before treatment. By helping identify high-risk polyps that may need wider resection or closer follow-up, the model supports more personalized treatment decisions and may reduce both under-treatment and over-treatment. Its use is expected to improve individual patient management and enhance the effectiveness of colorectal cancer prevention.

## 1 Introduction

Colorectal polyps are common lesions of the gastrointestinal tract, and their incidence continues to increase worldwide. These lesions are widely regarded as important precursors of colorectal cancer (CRC) (1, 2). CRC remains one of the most common malignant tumors globally and places a heavy burden on health care systems (2–6). Most CRCs develop through the classic adenoma–carcinoma sequence, which usually takes 5–10 years, providing a valuable window for early detection and intervention (7–9). Therefore, accurate pathological evaluation of colorectal polyps is essential for effective cancer prevention and appropriate treatment planning (10, 11).

In clinical practice, endoscopic forceps biopsy is still commonly used to assess the pathological features of colorectal polyps (12). However, biopsy samples often represent only a small portion of the lesion and may miss areas with more advanced pathology. Factors such as sampling error and pathological heterogeneity can lead to underestimation of the true histological grade (13, 14). This discrepancy may result in inappropriate clinical decisions, including insufficient treatment of high-risk lesions or unnecessary aggressive therapy for low-risk polyps. Although differences between preoperative biopsy and final pathology have been well described in gastric lesions (15–17), related evidence for colorectal polyps remains limited.

With the widespread use of high-definition endoscopy and image-enhanced endoscopy (IEE), current guidelines from leading endoscopic societies have changed clinical recommendations. For example, the Japanese Gastroenterological Endoscopy Society and the European Society of Gastrointestinal Endoscopy advise against routine forceps biopsy for many colorectal lesions, especially large non-pedunculated polyps, and recommend optical diagnosis based on high-quality endoscopic imaging instead. Despite these guidelines, endoscopic biopsy is still widely performed in daily practice to help evaluate polyp characteristics. In addition, even with advances in IEE and optical diagnosis, differences between endoscopic assessment and final histological diagnosis remain. This indicates that current endoscopic evaluation systems have inherent limitations and that further predictive factors are needed to improve diagnostic accuracy.

Recent studies have provided clearer evidence of this problem. Gorelik et al. (18) reported that, in large non-pedunculated colorectal polyps, the agreement between routine forceps biopsy and final pathology was only moderate, with a Kappa value of 0.55. Such limited consistency may increase uncertainty during clinical decision-making. To reduce the limitations of biopsy-based diagnosis, alternative strategies have been explored. However, as summarized in a recent systematic review by Gibiino et al. (19), approaches such as “resect and discard” are still not widely adopted. Their use is restricted by variability in endoscopic diagnosis and by legal and safety concerns for both clinicians and patients. As a result, treatment and follow-up decisions continue to rely heavily on histological results, while pre-treatment diagnostic tools remain imperfect.

Given these challenges, there is a clear need to better identify factors associated with pathological upgrading in colorectal polyps. Predictive models based on clinical and endoscopic features may help bridge the gap between biopsy results and final pathology. Traditional logistic regression models are widely used in medical research because of their stability and clinical interpretability. However, their results are often difficult to apply at the individual patient level. In recent years, explainable artificial intelligence methods, such as SHAP, have been introduced to improve model transparency by showing how each variable contributes to the predicted outcome (20).

Therefore, this study aimed to systematically evaluate the discrepancy between endoscopic forceps biopsy and postoperative pathological diagnosis in colorectal polyps and to identify independent risk factors for pathological upgrading. By developing and validating a prediction model and using SHAP to explain its results, we sought to provide a practical and interpretable tool to assist endoscopists in preoperative risk assessment and to support more accurate and personalized treatment decisions.

## 2 Materials and methods

### 2.1 Research design and participants

This prospective study enrolled 616 patients diagnosed with colorectal polyps by endoscopic forceps biopsy at the Fourth Affiliated Hospital of Anhui Medical University. All patients subsequently underwent endoscopic mucosal resection (EMR) or endoscopic submucosal dissection (ESD) between August 2022 and October 2025. Inclusion criteria were: (1) Biopsy showed an adenomatous polyp, followed by EMR or ESD; (2) confirmation that the same lesion was described in both biopsy and resection reports; and (3) availability of postoperative pathological results. Exclusion criteria included: (1) history of gastrointestinal malignancy; (2) Peutz–Jeghers syndrome; (3) familial polyposis; (4) Biopsy-confirmed hyperplastic polyp, inflammatory polyp, or other non-neoplastic polyps. (5) Biopsy performed by endoscopists with less than 3 years of independent experience or fewer than 200 procedures per year; and (6) an interval of more than 2 months between biopsy and resection. The study followed the Declaration of Helsinki and was approved by the Ethics Committee of the Fourth Affiliated Hospital of Anhui Medical University.

### 2.2 Clinical baseline data

Baseline data included age, sex, body mass index (BMI), smoking history, family history of colorectal cancer, and Cardio-Metabolic Syndrome (CMS). Laboratory data consisted of carcinoembryonic antigen (CEA) levels measured within 24 h of admission. Endoscopic features included maximum tumor diameter (MTD), morphology (pedunculated or sessile), number and location of lesions, surface color, presence of erosion, villous structure, number of biopsy samples, and bowel preparation quality. All procedures were performed using a high-definition endoscopy system (Olympus EVIS X1 with a CF-HQ190L colonoscope). Lesions were mainly assessed under white-light imaging.

### 2.3 Variable definitions and classification criteria

Polyp size was measured in millimeters and categorized using a 30-mm cutoff. This threshold was chosen for two reasons. First, prior studies and reviews frequently define polyps ≥30 mm as “giant” lesions and link them to higher malignant potential and the need for en bloc resection when feasible (21–23). For patients with multiple polyps, only the largest lesion based on MTD was included. Thus, 616 lesions from 616 patients were analyzed. Lesion location was classified as ascending colon, transverse colon, descending colon, sigmoid colon, or rectum. Surface color was categorized as near-normal mucosa or red based on endoscopic appearance. Bowel preparation quality was evaluated using the Boston Bowel Preparation Scale (BBPS) (24, 25). A total score of ≥6, with at least 2 points in each segment, was considered adequate. Endoscopists were not blinded to biopsy results, reflecting routine clinical practice. The endoscopist performing EMR/ESD did not blind the biopsy results. All resection specimens were independently reviewed by two experienced pathologists blinded to biopsy findings. Diagnoses followed the World Health Organization Classification of Tumours of the Digestive System (5th edition, 2019). Disagreements were resolved by a third senior pathologist. Pathological upgrading was defined as a higher pathological grade in the resected specimen compared with the biopsy result. Cases without such change were classified as no upgrading.

### 2.4 Statistical analysis

Statistical analyses were performed using SPSS 25.0 and R version 4.5.1. Continuous variables were expressed as mean ± standard deviation or median with interquartile range, depending on distribution, and compared using *t*-tests or Wilcoxon tests. Categorical variables were expressed as counts and percentages, and group differences were assessed using the chi-square test. Patients were randomly assigned to a training cohort and a testing cohort at a 7:3 ratio. The primary outcome was pathological upgrading after endoscopic resection, which was defined as a positive event. Feature selection was performed in the training set using least absolute shrinkage and selection operator (LASSO) logistic regression with the glmnet package. Predictors were automatically standardized. Ten-fold cross-validation was used to determine the optimal penalty parameter, and the 1-standard-error rule was applied to obtain a parsimonious model. The selected penalty parameter was *λ*_1se = 0.051818 (log λ = −2.960018). Variables with non-zero coefficients were retained for further analysis. A multivariable logistic regression model was then fitted, and a nomogram was developed. Model discrimination was evaluated using receiver operating characteristic (ROC) curves and the area under the curve (AUC). Calibration was assessed using calibration plots and the Hosmer–Lemeshow test. Decision curve analysis was performed to evaluate clinical utility. Model interpretation was conducted using SHAP analysis. Kernel SHAP was applied to estimate feature contributions, and results were visualized using summary and individual explanation plots. SHAP values were computed using up to 1,000 randomly sampled training observations with a background dataset of up to 100 training observations. Random sampling and fixed seeds were used to ensure stable results. The main R packages used included tableone, glmnet, rms, pROC, ResourceSelection, rmda, tidymodels, kernelshap, and shapviz. All tests were two-sided, and *p* < 0.05 was considered statistically significant.

## 3 Results

### 3.1 Overview of clinical and pathological characteristics in the cohort

In this prospective investigation, clinical and endoscopic information was gathered from a total of 616 individuals diagnosed with colorectal polyps. After 23 cases with incomplete data were excluded, 593 subjects were finally included in the statistical analysis. Based on the histological comparison between preoperative biopsy samples and specimens obtained after endoscopic resection, 150 patients were classified into the pathological upgrade group. The remaining 443 patients were placed in the non-upgrade group. The overall pathological upgrade rate was calculated to be 25.30%. A comparison of initial clinical and pathological indicators between the two groups revealed notable differences in lesion location, MTD, villous components, erosion presence, and surface characteristics. These statistically significant differences (*p* < 0.05) are detailed in Table 1.

**Table 1:** Overview of clinical and pathological data for all patients.

Variables	Total (*n* = 593)	Non-upgraded group (*n* = 443)	Upgraded group (*n* = 150)	*z*/*X*^2^	*p*-value
Gender [*n*(%)]				0.363	0.547
Female	86 (14.5%)	62 (14.0%)	24 (16.0%)		
Man	507 (85.5%)	381 (86.0%)	126 (84.0%)		
Age [*M*(Q1–Q3), years]	58 (50–71)	58 (51–71)	56 (50–71)	0.807	0.420
Smoking history [*n*(%)]				0.208	0.648
No	115 (19.4%)	84 (19.0%)	31 (20.7%)		
Yes	478 (80.6%)	359 (81.0%)	119 (79.3%)		
BMI [*n*(%)]				6.502	0.039
Normal	299 (50.4%)	212 (47.8%)	87 (58.0%)		
Underweight	187 (31.5%)	142 (32.1%)	45 (30.0%)		
Overweight	107 (18.1%)	89 (20.1%)	18 (12.0%)		
CEA [*n*(%)]				0.004	0.948
Negative	513 (86.5%)	383 (86.5%)	130 (86.7%)		
Positive	80 (13.5%)	60 (13.5%)	20 (13.3%)		
Family history of colorectal cancer [*n*(%)]				2.031	0.154
No	539 (90.9%)	407 (91.9%)	132 (88.0%)		
Yes	54 (9.1%)	36 (8.1%)	18 (12.0%)		
CMS [*n*(%)]				0.075	0.785
No	374 (63.1%)	278 (62.8%)	96 (64.0%)		
Yes	219 (36.9%)	165 (37.2%)	54 (36.0%)		
Maximum tumor diameter [*n*(%)]				60.021	<0.001
<30 mm	331 (55.8%)	288 (65.0%)	43 (28.7%)		
≥30 mm	262 (44.2%)	155 (35.0%)	107 (71.3%)		
Pedunculated tumor [*n*(%)]				0.580	0.446
Sessile	241 (40.6%)	184 (41.5%)	57 (38.0%)		
Pedunculated	352 (59.4%)	259 (58.5%)	93 (62.0%)		
Number of biopsy blocks [*n*(%)]				0.197	0.657
1 piece	483 (81.5%)	359 (81.0%)	124 (82.7%)		
≥2piece	110 (18.5%)	84 (19.0%)	26 (17.3%)		
Villi				43.499	<0.001
No	304 (51.3%)	262 (59.1%)	42 (28.0%)		
Yes	289 (48.7%)	181 (40.9%)	108 (72.0%)		
Surface [*n*(%)]				53.308	<0.001
Normal mucosal color	241 (40.6%)	218 (49.2%)	23 (15.3%)		
Red	352 (59.4%)	225 (50.8%)	127 (84.7%)		
Erosion [*n*(%)]				65.010	<0.001
No	348 (58.7%)	302 (68.2%)	46 (30.7%)		
Yes	245 (41.3%)	141 (31.8%)	104 (69.3%)		
Number of tumor [*n*(%)]				0.781	0.377
Single	418 (70.5%)	308 (69.5%)	110 (73.3%)		
Multiple	175 (29.5%)	135 (30.5%)	40 (26.7%)		
Intestinal cleanliness [*n*(%)]				3.115	0.078
Adequate Bowel Preparation	562 (94.8%)	424 (95.7%)	138 (92.0%)		
Inadequate Bowel Preparation	31 (5.2%)	19 (4.3%)	12 (8.0%)		
Location [*n*(%)]				144.913	<0.001
Ascending colon	73 (12.3%)	60 (13.5%)	13 (8.7%)		
Transverse colon	72 (12.1%)	63 (14.2%)	9 (6.0%)		
Descending colon	103 (17.4%)	95 (21.5%)	8 (5.3%)		
Sigmoid colon	142 (24.0%)	133 (30.0%)	9 (6.0%)		
Rectum	203 (34.2%)	92 (20.8%)	111 (74.0%)		

### 3.2 Comparison of baseline characteristics between training and test sets

Participants were randomly allocated to either the training or testing cohort in a 7:3 ratio, ensuring unbiased distribution. Their respective clinical profiles were analyzed according to this grouping. Specifically, the training group consisted of 415 patients diagnosed with colorectal polyps, among whom 104 cases (25.1%) demonstrated pathological upgrading. The testing group included 178 patients, with 46 individuals (25.8%) showing similar pathological progression. When all collected variables were compared, no significant differences were observed between the two cohorts (*p* > 0.05), suggesting a well-balanced baseline. Detailed information on baseline characteristics and corresponding statistical results is presented in Table 2.

**Table 2:** Comparison of baseline characteristics between training and test sets.

Variables	Train set (*n* = 415)	Test set (*n* = 178)	*P*-value
Gender [*n*(%)]			0.936
Female	61 (14.7%)	25 (14.0%)	
Man	354 (85.3%)	153 (86.0%)	
Age [*M*(Q1–Q3), years]	57 (50.0–70.0)	61 (51.2–73.0)	0.195
Smoking history [*n*(%)]			0.255
No	86 (20.7%)	29 (16.3%)	
Yes	329 (79.3%)	149 (83.7%)	
BMI [*n*(%)]			0.360
Normal	206 (49.6%)	93 (52.5%)	
Underweight	128 (30.8%)	59 (33.1%)	
Overweight	81 (19.5%)	26 (14.6%)	
CEA [*n*(%)]			1.000
Negative	359 (86.5%)	154 (86.5%)	
Positive	56 (13.5%)	24 (13.5%)	
Family history of colorectal cancer [*n*(%)]			0.928
No	378 (91.1%)	161 (90.4%)	
Yes	37 (8.9%)	17 (9.6%)	
CMS [*n*(%)]			0.608
No	265 (63.9%)	109 (61.2%)	
Yes	150 (36.1%)	69 (38.8%)	
Maximum tumor diameter [*n*(%)]			0.979
<30 mm	231 (55.7%)	100 (56.2%)	
≥30 mm	184 (44.3%)	78 (43.8%)	
Pedunculated tumor [*n*(%)]			0.107
No	178 (42.9%)	63 (35.4%)	
Yes	237 (57.1%)	115 (64.6%)	
Number of biopsy blocks [*n*(%)]			0.302
1 piece	343 (82.7%)	140 (78.7%)	
≥2piece	72 (17.3%)	38 (21.3%)	
Villus			0.347
No	207 (49.9%)	97 (54.5%)	
Yes	208 (50.1%)	81 (45.5%)	
Surface [*n*(%)]			0.604
Normal mucosal color	172 (41.4%)	69 (38.8%)	
Red	243 (58.6%)	109 (61.2%)	
Erosion [*n*(%)]			0.367
No	249 (60.0%)	99 (55.6%)	
Yes	166 (40.0%)	79 (44.4%)	
Number of tumor [*n*(%)]			0.323
Single	287 (69.2%)	131 (73.6%)	
Multiple	128 (30.8%)	47 (26.4%)	
Intestinal cleanliness [*n*(%)]			0.126
Adequate bowel preparation	389 (93.7%)	173 (97.2%)	
Inadequate bowel preparation	26 (6.3%)	5 (2.8%)	
Location [*n*(%)]			0.625
Ascending colon	55 (13.3%)	18 (10.1%)	
Transverse colon	51 (12.3%)	21 (11.8%)	
Descending colon	67 (16.1%)	36 (20.2%)	
Sigmoid colon	97 (23.4%)	45 (25.3%)	
Rectum	145 (34.9%)	58 (32.6%)	

### 3.3 Identification of predictive factors

**Figure 1:**
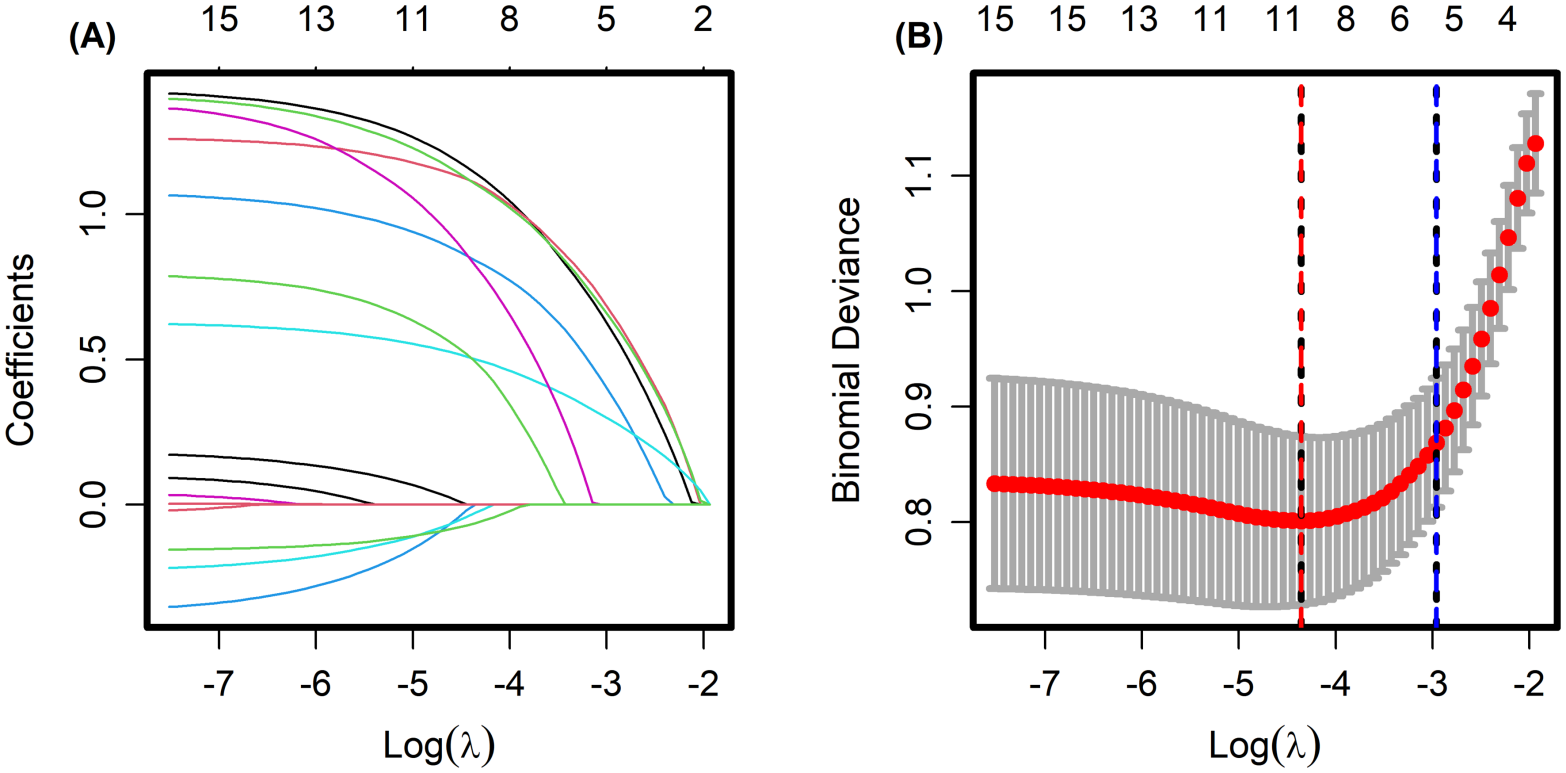
**(A)** Coefficient profiles of candidate predictors in the LASSO logistic regression model. Each curve represents the trajectory of a predictor coefficient as a function of the log(*λ*). **(B)** Ten-fold cross-validation curve for selecting the optimal penalty parameter (λ). The red dashed vertical line indicates the value of λ that minimizes the cross-validated error (λ_min), while the blue dashed vertical line represents the largest value of λ within one standard error of the minimum (λ_1se = 0.051818, log λ = −2.960018).

Patients were split into a training set (*n* = 415) and a testing set (*n* = 178) using the R function createDataPartition. LASSO regression was conducted in the training cohort to select variables with non-zero coefficients. With adjustment of the penalty parameter, the number of variables included in the model gradually declined. The model reached optimal performance with a *λ* value of 0.052 (logλ = −2.960) based on 10-fold cross-validation, as shown in Figures 1A,B. At this point, five predictive factors were identified: polyps located in the rectum, MTD ≥ 30 mm, polyps with a villous structure, polyps with erosion, and polyps with a red surface color.

### 3.4 Multivariate logistic regression analysis

As shown in Table 3, all five factors were independent predictors of pathological upgrading and showed statistical significance. Polyps in the rectum had the highest risk (OR = 6.58, 95% CI: 2.66–18.17, *p* < 0.001), meaning their risk was more than six times higher than that of polyps in other sites. Erosion was also strongly related to upgrading (OR = 4.36, 95% CI: 2.40–8.11, *p* < 0.001), indicating a much higher chance of underestimation by biopsy. Polyps with an MTD ≥ 30 mm, red surface color, and villous structure also had increased risks, with odds ratios above 2.8. These results showed that several endoscopic features had large effect sizes, not only statistical significance. In particular, rectal location and surface erosion were linked to a four- to six-fold higher risk, suggesting that such lesions require careful resection and closer pathological assessment.

**Table 3:** Multivariate logistic regression analysis of factors associated with pathological upgrading.

Variables	OR	95%CI	*P*-value
Location	6.58	2.66–18.17	<0.001
Erosion	4.36	2.40–8.11	<0.001
Maximum tumor diameter	4.10	2.26–7.67	<0.001
Surface	3.92	2.00–8.07	<0.001
Villi	2.89	1.57–5.46	<0.001

### 3.5 Model development and nomogram presentation

**Figure 2:**
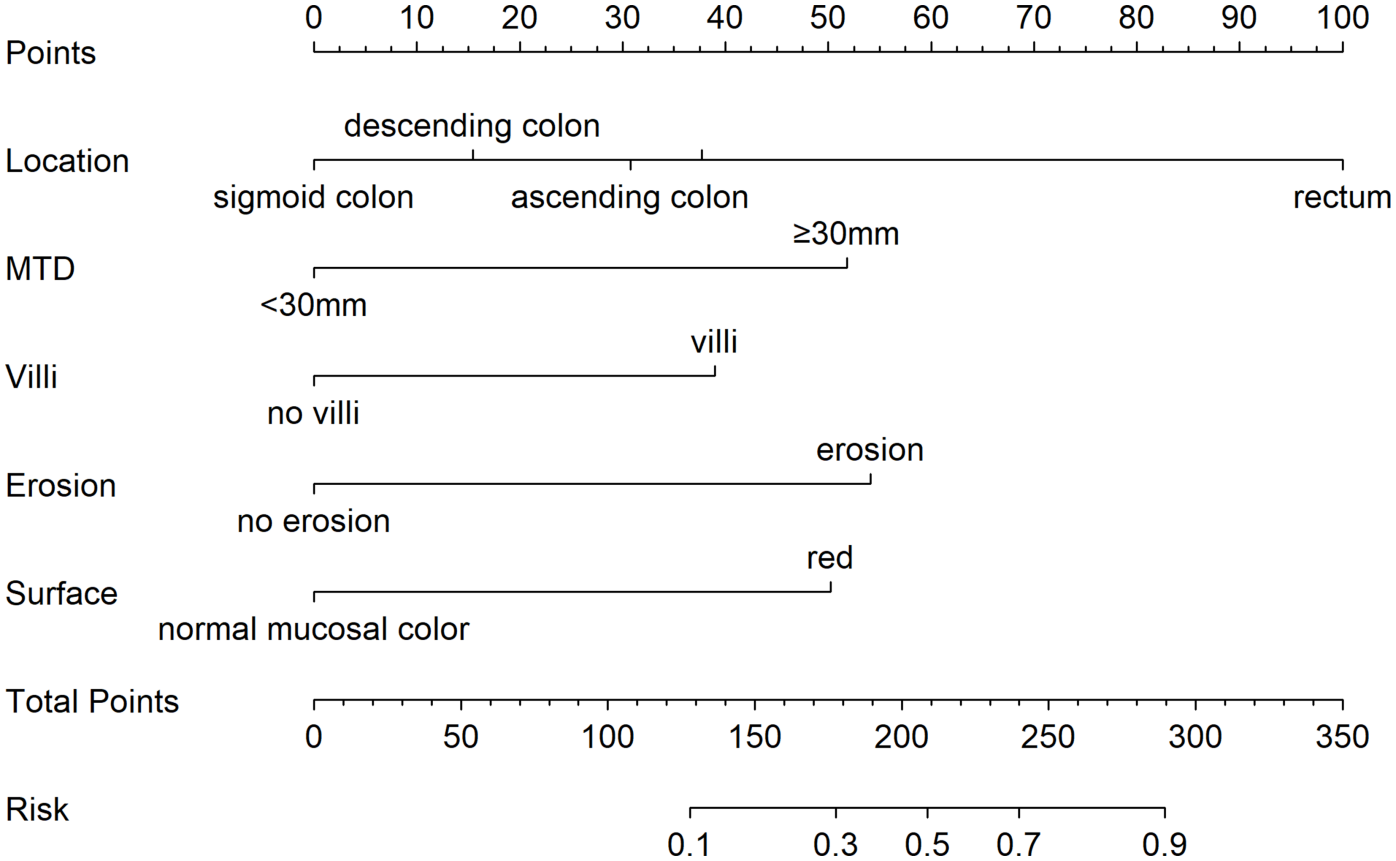
Nomogram for estimating the risk of pathological upgrading in colorectal polyp patients. The total points are calculated by summing the points for each predictor, and the corresponding predicted probability is obtained from the bottom scale. For example, a total score of 210 indicates a likelihood above 50%, while a score of 300 corresponds to a probability exceeding 95%.

Using five independent risk factors, we built a nomogram to estimate the risk of pathological upgrading in patients with colorectal polyps (Figure 2). The predictors included rectal site, MTD ≥ 30 mm, villous component, surface erosion, and red surface color. For each predictor, points were assigned and summed to obtain a total score, which corresponds to the estimated probability of pathological upgrading.

### 3.6 Model validation and clinical utility

**Figure 3:**
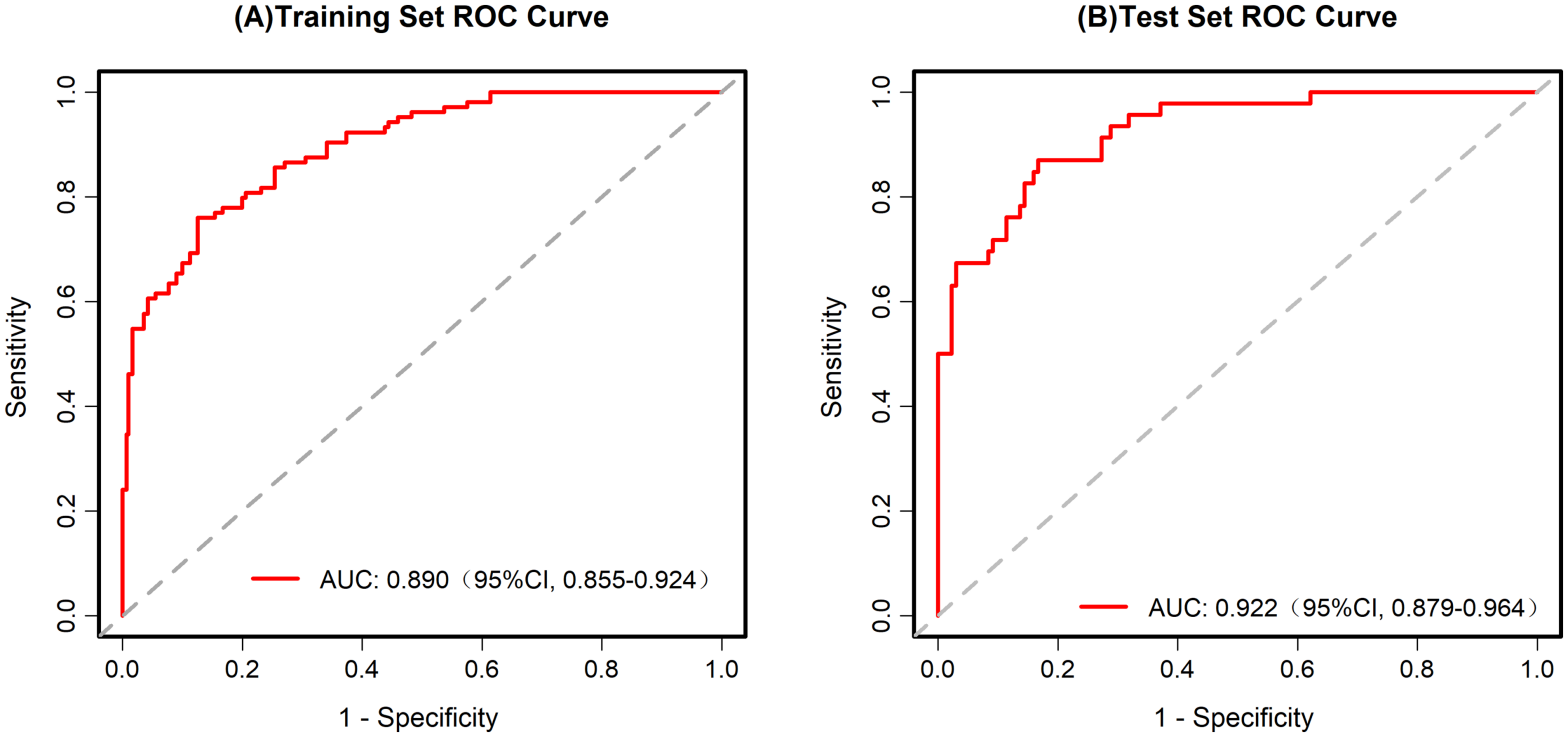
**(A)** ROC curve for the training set. AUC = 0.890 (95% CI: 0.855–0.924). **(B)** ROC curve for the test set. AUC = 0.922 (95% CI: 0.879–0.964).

Assessing the predictive capability of the scoring system for pathological upgrading in patients with colorectal polyps, this study used ROC analysis, calibration curve analysis, and DCA. For the training set, the AUC was 0.890 (95% CI, 0.855–0.924) (Figure 3A), while for the test set, the AUC was 0.922 (95% CI, 0.879–0.964) (Figure 3B). These AUC values demonstrate that the scoring system has strong diagnostic capability in both datasets.

**Figure 4:**
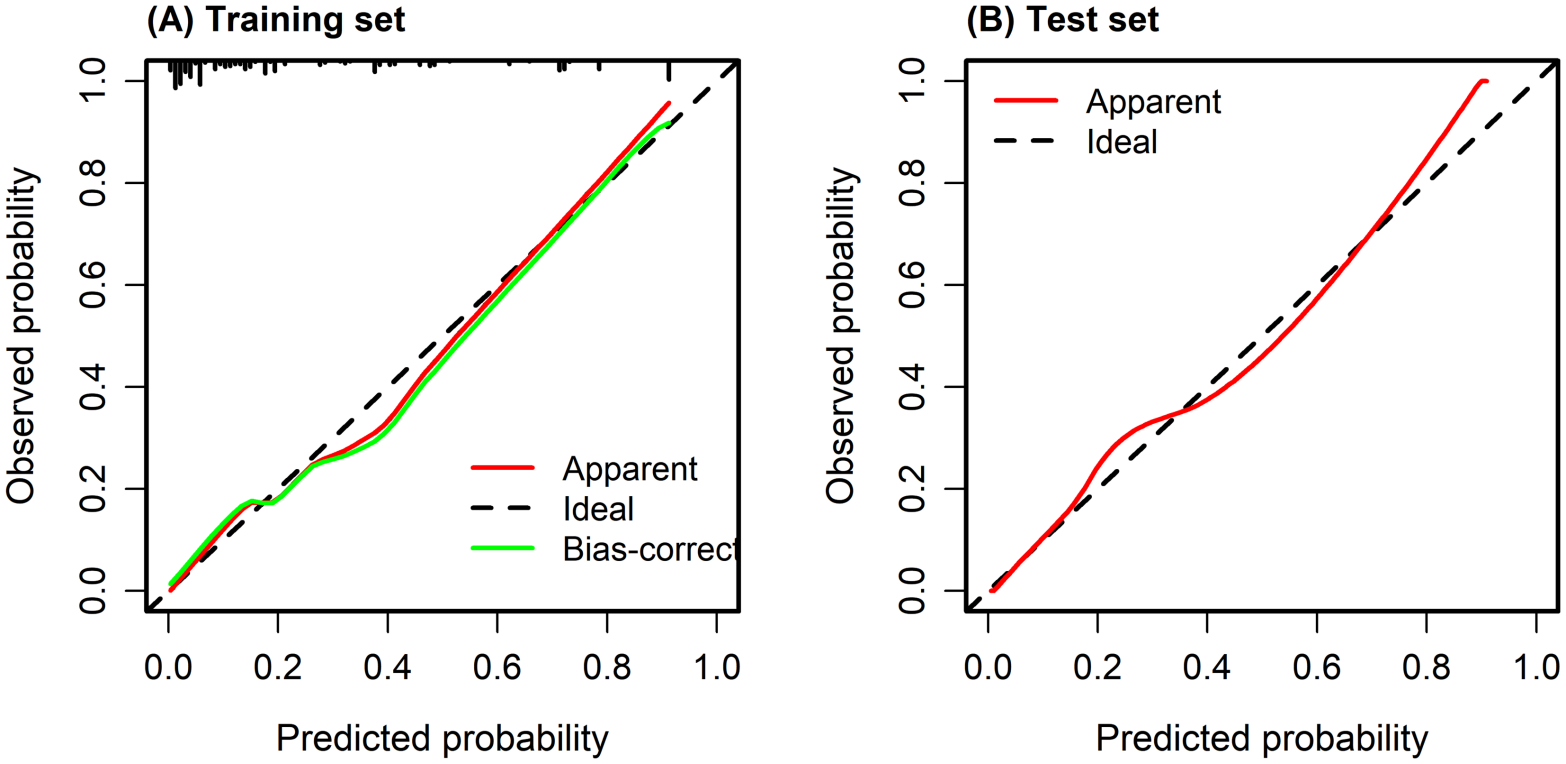
**(A)** Calibration curve of training set. **(B)** Calibration curve of test set. Calibration curves were used to evaluate the agreement between predicted probabilities and observed outcomes, with the diagonal line representing perfect calibration. The Hosmer–Lemeshow goodness-of-fit test showed no significant deviation between predicted and observed risks (*p* = 0.532), indicating good model calibration.

The fit of the model to the observed data was confirmed by the Hosmer–Lemeshow test (*p* > 0.05). The *X*-axis on the calibration curve represents the estimated probability of pathological upgrading in colorectal polyp patients, while the *Y*-axis indicates the probability that was actually observed. The ideal scenario, depicted by the diagonal line, is where the predicted values are the same as the observed values. When the calibration curve aligns closely with the diagonal, the model’s predictions are more accurate. As illustrated in Figures 4A,B, the calibration curve closely followed the reference line, indicating that the risk prediction model based on the scoring system is stable and dependable in clinical prediction.

**Figure 5:**
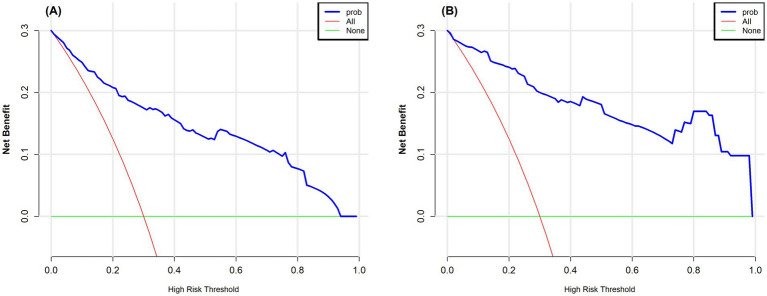
**(A)** Decision curve analysis for the training set. **(B)** Decision curve analysis for the test set. The vertical axis represents the net benefit, and the horizontal axis represents the threshold probability. The curve compares the “treat all” strategy with the “treat none” strategy, where a higher net benefit indicates better clinical utility. In the figure, the red curve corresponds to the “treat all” strategy, and the green curve corresponds to the “treat none” strategy.

This study also used DCA to assess the clinical usefulness of the model. Figures 5A,B show the DCA curves for both the training and validation datasets. In these figures, the *X*-axis indicates the threshold probability, while the *Y*-axis represents the net benefit. The green curve serves as the reference line, showing the outcome when no intervention is given. In contrast, the red curve reflects the net benefit when every patient receives the intervention. The blue curve remains above the reference line across a threshold probability range of 0.1–0.9, suggesting that the model offers a clear net benefit in most situations. These results support the reliability of the model and demonstrate its value in clinical practice.

### 3.7 Explainable AI analysis with SHAP

**Figure 6:**
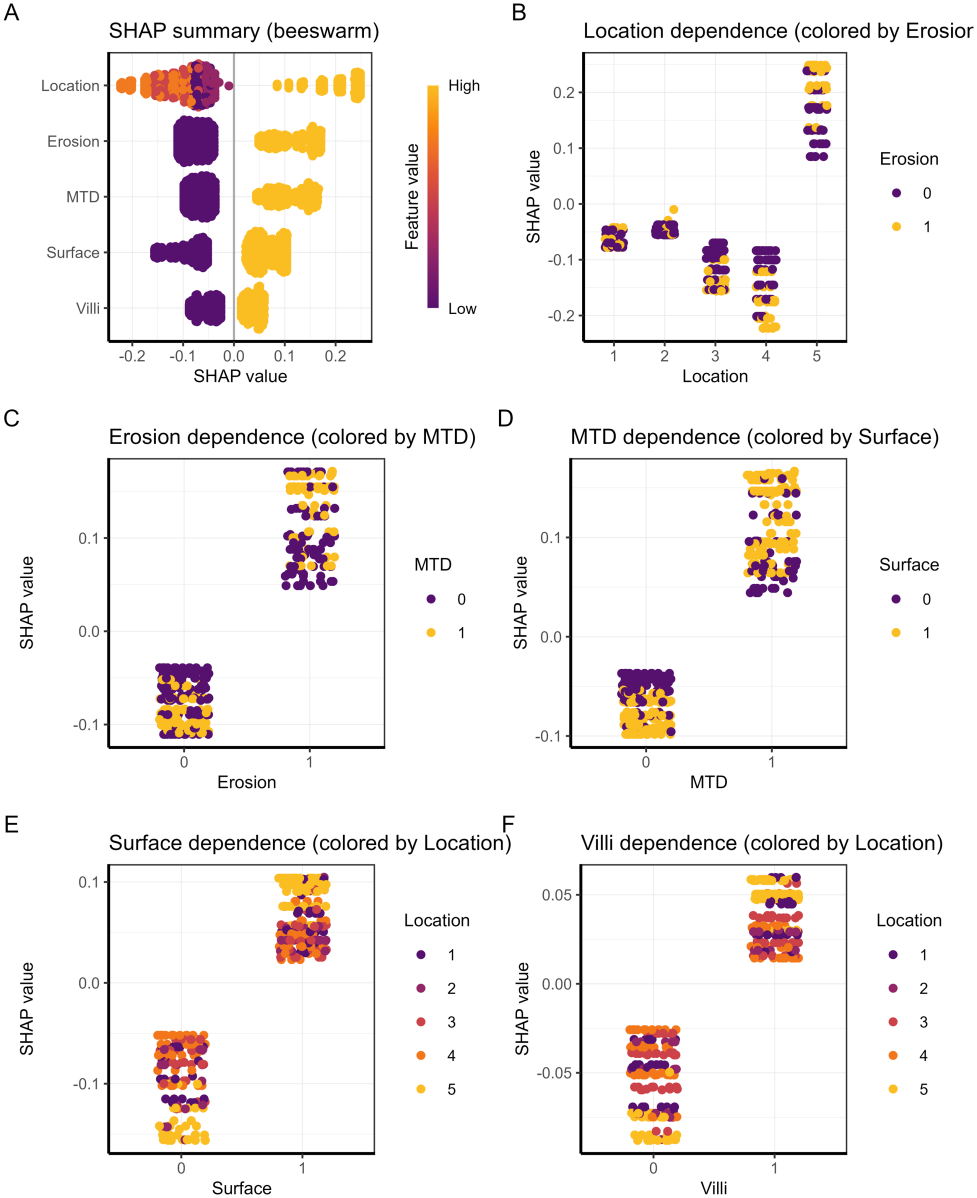
**(A)** Summary plot of SHAP values. Each dot represents a patient, and the color indicates the feature value. **(B–F)** SHAP dependence plots illustrating the relationship between each predictor and its SHAP value for **(B)** location, **(C)** erosion, **(D)** MTD, **(E)** surface, and **(F)** villi.

To develop an effective prediction model, it is crucial to accurately identify the key factors affecting the pathological upgrading of colorectal polyps. Applying statistical approaches to compare these features can enhance both the predictive performance and interpretability of the model. In this study, SHAP analysis was used to visually examine the contribution and reliability of the selected predictors. The results indicated that five variables were closely associated with pathological upgrading: rectal location of the polyp, MTD ≥ 30 mm, villous pattern, erosive morphology, and red surface appearance. As shown in Figure 6A, each endoscopic feature is significantly related to the risk of pathological classification, among which location characteristics are the strongest predictor, followed by erosion. Figures 6B–F shows that positional characteristics, as the most important predictor, exhibit obvious site-specific risk values, among which the risk of rectal progression is the highest. Erosion is closely related to increased risk and interacts with the MTD—the larger the lesion area, the more significant the erosion effect. The presence of erythema on the surface of polyps increases the overall risk, but the effect is influenced by the site: in areas such as the rectum, the risk of increased pathological grading is more prominent. The villous structure is also positively correlated with risk and shows heterogeneity across different anatomical sites, with the risk increase in the rectal region being the greatest. The complex interaction among these characteristics together constitutes a multifactorial prediction model for disease progression.

**Figure 7:**
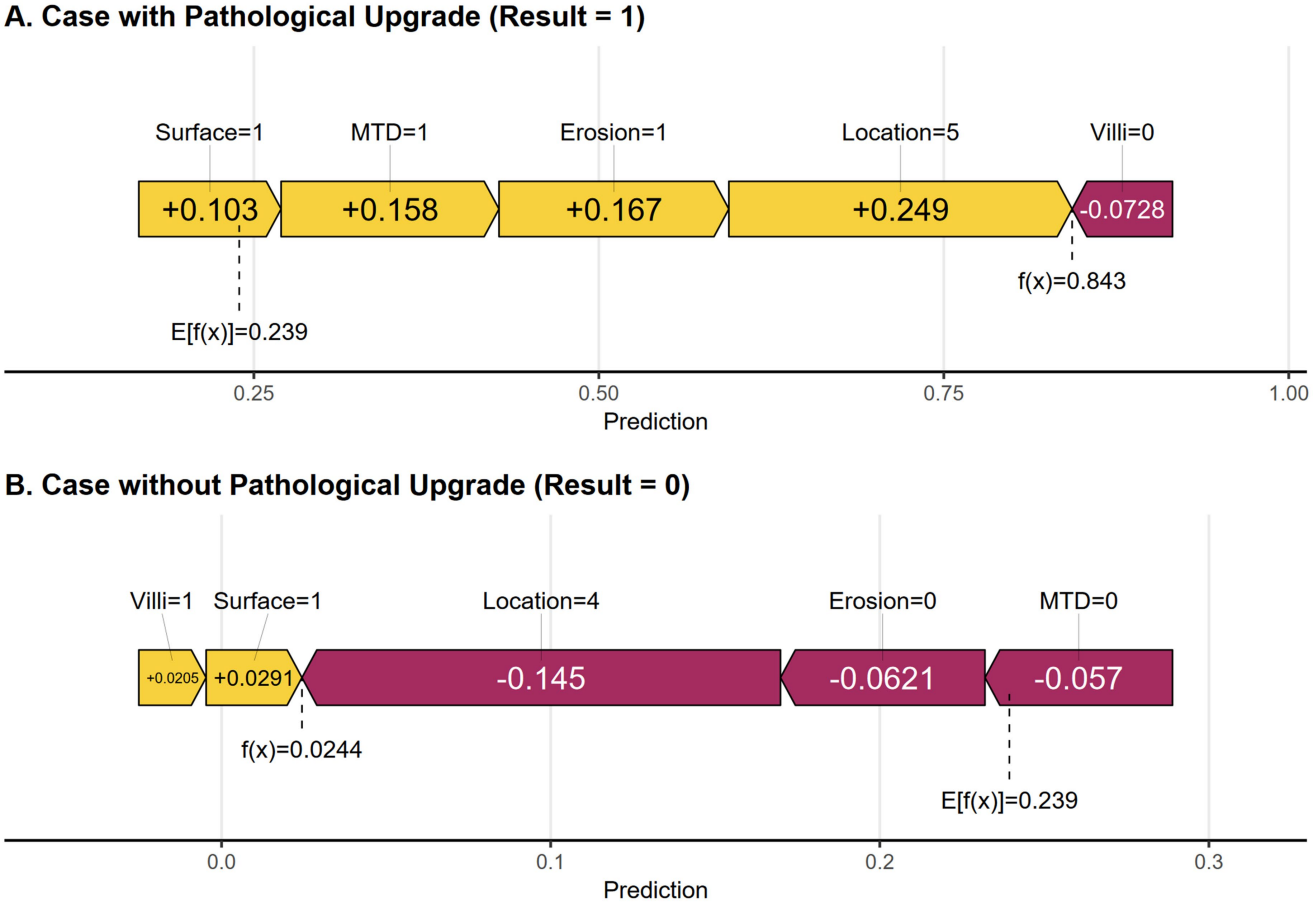
**(A)** SHAP force plot of a positive result cases. **(B)** SHAP force plot of a negative result case. Yellow features increase the predicted probability, while purple features decrease the predicted probability.

Two examples are provided to explain the effect of each variable on individual sample predictions. As shown in Figure 7A, the model predicted a positive outcome for a colorectal polyp patient who indeed experienced an upgrade in pathology. The predicted probability for this high-risk case is 84.3%, indicating that factors such as rectal site, erosion, maximum tumor diameter ≥30 mm, and red surface all have a positive impact on the likelihood of pathological grade increase. Together, these factors significantly increase the predicted probability from the baseline value of 23.9%, with the rectal site contributing the most (+0.249). In contrast, Figure 7B shows that the predicted probability for low-risk cases is 2.4%, among which protective characteristics (such as absence of erosion, non-rectal site, and MTD < 30 mm) have a significant negative impact. The risk reduction effect of non-rectal sites is the most significant (−0.145). These individual-level interpretations not only verify the rationality of the model but also clarify the quantitative contribution of key risk factors.

## 4 Discussion

Colorectal polyps represent a serious burden to health care, as they affect long-term outcomes and reduce the quality of life in many patients. The incidence and mortality of colorectal cancer are still increasing worldwide, and colorectal polyps have been identified as important precursor lesions of this malignancy (26–28). Therefore, it is very important to carry out accurate risk stratification of colorectal polyps. Although biopsy-guided treatment is widely used, it often leads to misjudgement of lesion grading due to sampling errors and differences in diagnosis. The resulting clinical problems include the risk of inadequate treatment (such as incomplete removal of lesions that may have highly differentiated characteristics) and the risk of overtreatment (radical strategies for actually low-risk lesions) (14, 29). In view of these challenges, the development of a risk model that can accurately predict the graded escalation of pre-treatment pathology is of great clinical value. This kind of model will help to achieve more personalized and accurate colorectal polyp management.

This study analyzed 593 patients. In univariate analysis, five endoscopic features—rectal location, MTD ≥ 30 mm, villous pattern, erosion, and red surface color—were significantly related to pathological upgrading (*p* < 0.05). These factors were repeatedly selected by LASSO regression and remained independent predictors in the multivariable logistic model. The final model showed good discrimination and calibration, as supported by ROC curves, calibration plots, and decision curve analysis. In addition, SHAP analysis further explained the impact of each predictor. Overall, these results emphasize the important role of lesion morphology in identifying patients at high risk of pathological upgrading.

Previous studies have shown that lesion size and location are key factors influencing the difference between biopsy and postoperative pathology (14). A Korean study reported that polyps ≥10 mm were more likely to show pathological upgrading after resection (30). In clinical practice and predictive models, a 30-mm cutoff is commonly used, as lesions of this size are generally considered large and are often associated with malignant potential (21–23). In our study, polyps ≥30 mm had a 4.61-fold higher risk of pathological upgrading. One possible reason is that larger lesions have more complex and uneven pathology. Biopsy samples represent only a small part of the lesion, and this proportion decreases as lesion size increases, leading to underestimation. In addition, larger polyps carry a higher risk of malignancy (14). Together, these findings support MTD as an independent risk factor for pathological upgrading in colorectal polyps.

This study showed that polyp location and surface color were important risk factors for pathological upgrading. Polyps located in the rectum had a higher risk of upgrading than those in other colonic segments, which was consistent with previous reports (30–32). This may be related to anatomical features of the rectum that limit representative biopsy sampling and increase the chance of pathological underestimation before resection. We also found that red surface color was strongly associated with pathological upgrading (33). Zhang et al. (37) reported that surface hyperemia was linked to advanced pathology, with an odds ratio of 3.5 (95% CI: 1.25–9.82). Compared with their findings, red surface color in our study showed a stronger predictive effect and remained an independent predictor after multivariable adjustment. This difference may be related to improved endoscopic observation and more detailed feature assessment. These results support the value of erythema in preoperative risk stratification and clinical decision-making.

Similar to previous studies, we found that both surface erosion and villous-like structures of polyps were strongly associated with pathological upgrading. Polyps with surface erosion often show tissue disruption and reduced mucosal integrity during endoscopy, and these features are commonly viewed as warning signs of lesion progression. In this study, the pathological upgrade rate was significantly higher in erosive polyps than in non-erosive ones (22, 31, 34). Villous structures also serve as important predictors of pathological upgrading. Villous polyps usually present with a raised surface and densely arranged, uneven glands, and these morphological features are often linked to a higher pathological grade. Several clinical studies have confirmed that adenomas containing villous components ≥25% have a higher likelihood of developing high-grade intraepithelial neoplasia or early-stage carcinoma (13, 35). The results of this study further support this pattern.

Unlike most retrospective studies, this study used a prospective design to collect clinical and endoscopic data, which improved data quality and reduced bias. The prediction model was based on routine features, including polyp size, location, erosion, villous structure, and surface color, making it practical for preoperative risk assessment. To improve model interpretability, logistic regression was combined with the SHAP method. SHAP clearly showed how each endoscopic feature contributed to the risk of pathological upgrading, allowing endoscopists to better understand the model results and compare them with their own experience. This approach is both methodologically innovative and clinically meaningful, as it links statistical prediction with real endoscopic findings. Importantly, SHAP-based explanations supported clinical decisions. High-risk features, such as rectal location, large size, or erosion, suggested the need for more careful resection or pathological evaluation, while low-risk results helped avoid unnecessary aggressive treatment. Overall, the use of SHAP improved both the transparency and clinical value of the prediction model (36).

Some limitations remain in the present study. First, this was a single-center, prospective study. Critically, the lack of independent external validation curtails the clinical interpretability and generalizability of our findings. While internal validation demonstrated good discrimination, performance is intrinsically linked to our center’s specific demographic, endoscopic, and pathological protocols. We cannot quantify the potential performance decay (e.g., in AUC or calibration) in broader practice, which profoundly affects the model’s readiness for deployment across institutions with differing patient populations, technology, or reporting standards. Therefore, our results must be considered preliminary and center-specific. Second, endoscopists were not blinded to the pre-resection biopsy results, which reflects routine clinical practice but may introduce information bias and potentially influence the endoscopic assessment and reporting of lesion features. Future studies should consider blinded central review of endoscopic images or videos by independent endoscopists who are unaware of pre-resection biopsy results, using standardized scoring forms. Third, the main outcome of this study was the agreement between preoperative biopsy results and immediate postoperative pathology. Long-term follow-up data after surgery were not available. As a result, the relationship between the model predictions and long-term outcomes, such as polyp recurrence, metachronous tumors, or other clinical events, could not be evaluated. This limits the value of the model in predicting the long-term biological behavior of lesions. Fourth, the rate of pathological upgrading in this cohort was approximately 25%, indicating a moderate degree of class imbalance. Such imbalance may affect model stability and calibration, potentially leading to optimistic discrimination metrics. Although this distribution reflects real-world clinical practice, no specific resampling or cost-sensitive learning techniques (e.g., SMOTE or class weighting) were applied. To mitigate overfitting and instability, LASSO regression was employed for variable selection, and model performance was comprehensively assessed using discrimination, calibration, decision curve analysis, and SHAP-based interpretability. Nevertheless, further validation in larger and more balanced datasets is warranted to confirm the robustness of the model. Finally, while LASSO regression helps reduce multicollinearity during feature selection, formal collinearity diagnostics were not performed, and residual collinearity effects cannot be entirely excluded. Future multicenter studies with larger sample sizes, external validation cohorts, and long-term follow-up are needed to further refine and validate the model across diverse clinical settings.

## 5 Conclusion

The study formulated a risk prediction model for pathological upgrading in colorectal polyps, incorporating five essential predictors: rectal location, MTD ≥ 30 mm, villous structure, erosion, and red surface color. The model is presented in the form of a nomogram to facilitate clinical application. By helping clinicians identify high-risk patients before surgery, the tool supports the formulation of more personalized treatment plans and is expected to reduce the incidence and mortality of colorectal cancer through timely intervention. Special attention should be paid to rectal lesions, which are often difficult to assess and are easily underestimated by pathology. Therefore, detailed preoperative evaluation and excision strategies must be formulated.

## Data Availability

The raw data supporting the conclusions of this article will be made available by the authors, without undue reservation.
